# Using Communities of Practice Theory to Understand the Crisis of Identity in Chronic Fatigue Syndrome/Myalgic Encephalomyelitis (CFS/ME)

**DOI:** 10.1177/17423953211064989

**Published:** 2021-12-06

**Authors:** Rebecca Murray, Lynda Turner

**Affiliations:** 4013 The University of Huddersfield, Huddersfield, UK

**Keywords:** CFS/ME, identity, virtual communities, meaningful participation

## Abstract

**Objective:**

To explore the crisis of identity in Chronic Fatigue Syndrome/Myalgic Encephalomyelitis (CFS/ME) through the lens of Communities of Practice.

**Methods:**

A closed Facebook group was created to gather qualitative data from participants diagnosed with CFS/ME (n = 22). Data were analysed using a theoretical thematic analysis.

**Results:**

The current research revealed the reality of enabling and disabling communities in the lived experience of CFS/ME and the role of participation in developing empowered identities. Learning how to be alongside CFS/ME aligned with participants’ experiences of purpose and meaning. New identities may be developed which are not centrally defined by loss or stigma.

**Discussion:**

Participation in supportive communities enables CFS/ME identities to emerge as a platform for positive change. Engaging with the CFS/ME virtual community may be a way for both families and health professionals to reflect on current practice.

## Introduction

Chronic Fatigue Syndrome (CFS) also known as Myalgic Encephalomyelitis (ME) is a chronic illness which is primarily defined by severe, incapacitating, and often punishing fatigue of new onset and has been present for at least 6 months.^1,2^ The symptom profile is complex and as such alongside the predominant symptom of chronic fatigue, there are a wealth of additional symptoms, such as muscle pain and weakness, headaches, fever, sore throat, and cognitive dysfunction such as impaired memory and sleep disturbance.^3^ The severity of CFS/ME can vary, however those with the condition are often unable to sustain their occupational, educational, and/or social roles.^2^

Although the prevalence of CFS/ME in the UK is estimated to be 0.2–0.4%^4^ CFS/ME predominantly affects women. As biomedical evidence remains elusive, CFS/ME is a controversial chronic illness that is burdened by the history of hysteria, scepticism and disbelief.^5^ Despite being a gendered illness with an alleged lack of biomedical evidence, ME remains classified by the WHO (1969) as a neurological disorder in the International Classification of Diseases (ICD).^6^ The lack of biomedical evidence caused much controversy in the 1980's which resulted in a reclassification. Chronic Fatigue Syndrome (CFS) was constructed in an attempt to enable scientific research into ME, but in so doing obliterated fundamental features of ME.^7,8^ CFS has since been classified by the WHO (ICD-10) in Chapter V ‘Mental and Behavioural Disorders’ under 48.0 ‘Other Neurotic Disorders’.^6^

In 1998 a Working Group of experts in the field of CFS and ME collaborated with the National Health Service (NHS) to establish how people with CFS and ME could be best supported by the NHS. Despite the Working Group (2002) recommending the CFS/ME acronym to diffuse the discontent amongst the ME community, CFS diagnoses continue to take precedence. Such diagnoses exasperate those who believe they are symptomatic of neurological ME, not psychosomatic CFS as primary care remains a hostile and unsupportive territory for those who present with CFS/ME.^4,9–14^ This lack of support within primary care is particularly challenging as currently there is no cure. Thus, the impact of CFS/ME is life changing. According to the literature, CFS/ME is a multifaceted assault on both life and self.^3,15,16^

### CFS/ME and identity literature

There is an existing body of literature which considers the issues of identity, which are central to the lived experience of CFS/ME. The biographical disruption caused by CFS/ME and experiences of identity transformation have been explored.^3,16–20^ The relationship between the jeopardised identity of those with CFS/ME and the contested nature of the illness has also received attention.^21–23^ Although such studies report the experiences of participants, they typically do so without attempting to understand the processes of identity change theoretically. When both life and self are unrecognisable in chronic illness, successful coping involves both acceptance and adjustment.^23^ To adapt to a life and self with chronic illness we need to better understand the processes involved.

## Our theoretical lens

Identity is an individualised experience as we each have a unique understanding of what it means to occupy our various roles, but it is also something that others confer on us. Identity can therefore be collective, for example the descriptor “woman” or “patient” conjure up a set of expectations, assumptions and actions connected to being a member of these groups. Identity is something we are actively engaged with but also something that others do to us. How we perceive ourselves will change depending on where we are, who we are with and how we are treated. The notion of multiple identities and trajectories across time and place is a central theme in Communities of Practice theory (CoP),^24^ which we employ in this paper to explore living with chronic illness. In applying CoP to the issues of identity in CFS/ME we aim to explore the mechanisms underpinning jeopardised and renegotiated identities.^25^

## Participation and identity

CoP is a social theory of learning which is most widely used in transition research. A diagnosis of chronic illness arguably requires the management of change which suggests the potential for the application of CoP. The underlying assumption of CoP is that learning and identity should not be mistaken as solely independent pursuits. Lave and Wenger (1991) discussed the ways in which participation shaped identity through the example of membership in Alcoholics Anonymous (AA). When a person joins AA they attend regular meetings, hearing the testimonies of old timers in the community. Over time, their participation changes, as they are encouraged to share their own life story and adopt the Twelve Steps practices to sobriety of A.A. They journey from the periphery of the community to full participation and in doing so develop a new identity of recovering alcoholic. Participation provides the opportunity to renegotiate a stigmatised identity. As a full participant they move from apprentice to expert and in turn can offer support to newcomers. As the mechanism underpinning identity is explicitly claimed to be participation, CoP provides a concise theoretical framework within which to consider the chronic illness identity trajectory of people living with CFS/ME.

In CoP theory, participation reflects the experiences of purpose and meaning which nurture identity such as the activities which one participates in, for example employment, and hobbies, and the roles which one embodies, for example student, and Mother.^25^ In chronic illness, participation becomes problematic. The activities and roles which once defined you are no longer available. Unlike the identity change which comes with single life transitions, such as changing jobs or retiring, chronic illness requires adjustment across numerous communities and identities.

## The concept of reification

Acquiring the label of chronic illness also involves engagement with medical professionals and their reified practices. When we reify a phenomenon, we turn experience (for example “feeling unwell”) into material objects (for example “treatment strategies” or “diagnosis”). This becomes a focus for the negotiation of meaning and efficient shared practice in a community. However, Wenger also argues that the focusing strength of reification can prohibit new ideas and lead to a disconnection from the lived experience. In illness, reification can involve definitions which contribute towards a legitimate sick identity. However, within contentious chronic illnesses such as CFS/ME, sick identities may be denied if stigmatised identities are reified. Applying CoP to the experience of chronic illness would therefore allow for a theoretical exploration of identity negotiation and participation in reified medical practice. It may also afford a means of professional reflection on taken for granted ways of being without blaming individual practitioners.

## Enabling and disabling practices

Communities of practice are woven into the tapestry of life and self. The activities and roles which underpin participation are indicative of community membership which engenders a sense of knowing; this is who I am. However, community membership is not rigid, and thus members traverse community boundaries as they negotiate their evolving identities.^25^ The potential to participate in communities of practice is jeopardised by the impact of chronic illness which often realises itself in experiences of unavoidable withdrawal and isolation. Communities can be both enabling and disabling. Enabling communities of practice are emerging structures which rest on a foundation of development and change. Learning within communities can be collaborative and innovative within the context of old and new. Changes in an individual member, due to chronic illness, could be negotiated by the community through adapting practice to support continued participation. This might include changes to working hours, access to technological support or a renegotiation of family roles. It also suggests the potential for the development of new communities such as support groups. Communities can also engage in disabling practices which prohibit engagement. CoP then allows for an understanding of identity through participation and provides an analytical focus which considers the individual and the communities they inhabit.^25^ In this paper we aim to explore the crisis of identity in CFS/ME through the lens of CoP.

## Methodology

The current article draws upon data from “A life lived differently: An exploration of how living with Chronic Fatigue Syndrome/Myalgic Encephalomyelitis (CFS/ME) impacts upon people's identity” (Unpublished doctoral dissertation, The University of Huddersfield). Participants diagnosed with CFS/ME were recruited from publicly accessible CFS/ME Facebook groups and invited to join a research group which signified informed consent. Participants’ data (n = 22) was subsequently gathered via a closed Facebook group called ‘cfsid’ which was an amalgamation of CFS and identity (ID), for people with CFS/ME. cfsid was created for the purpose of research and moderated by the researcher. Over an approximate six month period in 2014, participants initially completed preliminary activities. The ‘Timeline’ activity asked participants to share a few details about certain times in their life: life prior to becoming ill; life on becoming ill; life since diagnosis; and, life now. The ‘Twenty Statement Test’ (TST) activity required participants’ to describe their illness journey by considering categories such as physical characteristics; roles; emotions; activities; whilst also considering the future; hopes and fears. Such consideration was guided by a predetermined timeline: I am; I was; and, I would like to be. The TST endeavoured to illuminate the shifts which had occurred in participants’ lives since the onset of CFS/ME. Participants subsequently engaged in various conversations surrounding their lived experience of CFS/ME. Some were started by the researcher whilst others were started by participants. As a person who has had CFS/ME, I had a type of insider role within the research and was able to generate and facilitate discussions surrounding the lived experience of CFS/ME. The research design endeavoured to enable the voices of participants and in so doing enable their ‘truthful’ CFS/ME and identity stories to emerge longitudinally. This decision was driven by my knowledge of the CFS/ME community and their need for ‘time’. Participants were able to participate on their own terms, as and when they were able to. As such, participants were able to break down the activities, return to activities, pause conversations until a better day arrived, voluntarily return to edit their contributions, and elaborate upon certain contributions at the request of the researcher. The research was therefore designed with enabling practices in mind to maximise engagement and participation. Comprehensive stories of jeopardised identities in CFS/ME emerged within the longitudinal data. An insider researcher cannot deny that their own lived experience of the topic may have an impact on the data collection and analysis.^26,27^ As such, reflexivity was central to the data analysis. A combination of time lapse reviews whereby the insider researcher returns to their analysis after approximately two weeks to view the data through a ‘new lens’ and peer consultation can illuminate possible projections and/or content which has been overlooked by the insider researcher. For the purpose of the current paper, analysis was undertaken collaboratively by both authors with different positions, insider and outsider. This allowed the insider researcher to communicate their interpretations which made transparent any potential bias in interpretation. Time lapse reviews also enabled both authors to reflect upon and reconsider their interpretations before findings were agreed.^28^ As the application of Communities of Practice (CoP) theory underpinned the doctoral research, here the preliminary activity and conversational data were analysed jointly for the purpose of the current paper using a theoretical thematic analysis.^25,29^ We interrogated the data using the key concepts of CoP: participation (or non-participation) in community membership; trajectory in identity; reification; enabling and disabling communities and practices. We used the ‘Standards for Reporting Qualitative Research’ (SRQR) to establish the requirements elements of reporting such work.^30^ The researchers’ university's Ethics Committee (SREP) approved the study. As an insider researcher I understood the need to draw the research to a close sensitively. The purpose of cfsid being research was made transparent from the start, as was the predicted timeframe of 6 months. However, following data collection and analysis, on seeing a community develop it was agreed that cfsid would remain active for research updates and to allow friendships and community support to continue within the safety of the group.

## Thematic analysis

The research revealed a number of themes: Loss of communities and identities; Interrupted futures; Living with disbelief; and Finding community.

## Loss of communities and identities

According to the literature, biographical disruption appears central to the lived experience of chronic illness: CFS/ME.^3^ Feelings of loss and grief aligned with the shift in peoples’ lives from active to passive, and their inability to sustain their previous life roles, which jeopardised their identities.^21^ Similarly, an inability to sustain the roles, and behaviours which were once central to a sense of self caused a fundamental shift in their identities.^20^ The findings of the current research also demonstrate biographical disruption explored using the application of CoP. All the practices and communities, which made participants ‘who they were’, prior to the onset of CFS/ME were now historical. Participants were no longer able to ‘be’ as they were no longer able to ‘do’. The following quotes reveal the impact of this loss:

My life was full until it stopped…I felt like a non person (Olivia)

I was a doer, involved in anything and everything… all that stopped when I became ill and now I’m not sure who I am. (Kate)

Inability to participate in their social communities has a profound influence on identities which must exacerbate the distress caused by the symptoms of illness itself.

## Interrupted futures

Wenger conceptualised identity and participation as trajectories. Our journeys through and across communities are often outward focused. For example, we may participate fully in a learning community as it offers a potential transition into a desired profession. For our participants, these future trajectories were also lost Whilst the following quotes reflect biographical disruption, they also reveal the reality of interrupted future trajectories in the lived experience of CFS/ME:

I decided not to pursue a relationship or have children (Jessica)

I do not recognise myself, and I do not recognise my life. I honestly don't believe that this life was meant for me. I really don't think that this is the life that I am supposed to be living. (Olivia)

When trajectories are interrupted as in the case of CFS/ME, futures become uncertain. Not only are identities jeopardised in the present but they are also sabotaged within future expectations of self.

## Living with disbelief

Previous research has considered how scepticism surrounding the illness contributed to a sense of isolation and stigma.^16^ We argue that participants’ jeopardised identities were compounded by their inability to adopt the ‘sick role’ due to the scepticism amongst health professionals surrounding CFS. The history of the illness and medical scepticism meant that participants were marginalised in the very community which was tasked with supporting them:

I didn't feel anyone believed me. I was passed from one consultant to another (Erin)

My GP said it was all in my head (Dan)

My current GP practice does not believe in acronyms including ME/CFS/IBS… One GP said if I held a gun to your head would you go to work… and that if I lived in the 3^rd^ world I would have no choice but to work. (Jessica)

The illegitimate and psychosomatic nature of the illness appear to be reified in medical communities, meaning participants were not privileged with a sick identity which compromised their potential to ‘be’ alongside CFS/ME. Participating in CFS/ME may not appear to be a useful form of participation, however, participating in an illness has the potential to enable a new identity to emerge, a sick identity which can legitimise an illness experience and engender the acceptance and support of others. Within the current research, primary care was seen as a disabling community. Meaningful participation was denied which we argue has limiting consequences not only for the individual but also for the development of supporting practices in the medical profession. In failing to legitimise patients’ experiences, the medical community practices remain unchanged. Reification is prohibiting transformation in the community and disabling practices continue.

Primary care was not the only disabling community our participants experienced:

I’ve had and still have from some of my family total disbelief and refusal to accept or understand my illness… My parents have said that I am no longer their daughter… They have said it`s all in my mind and that I am lazy!!!! (Ros)

As well as the medical construction of CFS/ME as a psychosomatic illness, family and friends may also respond with disbelief and stigmatisation. These familiar communities have the potential in chronic illness to support and thus enable meaningful participation, however our data suggested that for some participants they were experienced as disabling communities. Familial identity is disrupted in chronic illness. This may exacerbate an already debilitating condition and suggests that support should be offered to families in negotiating new practices so that family participation can evolve.

## Finding community

Despite a widespread sense of isolation, the data also revealed participants’ renegotiation of life and self through a virtual connection to others. Accessible online communities can be a great source of support for those who live alongside chronic illness and disability. In recent years there has been a significant increase in people turning to the internet for information.^29,31,32^ In chronic illness and disability, online platforms such as Facebook can be inspirational as they enable others to see what may be possible within their lived experience of chronic illness and disability.^29,31,32^ Facebook group membership was an enabling community as it offered familiarity, acceptance, and support, whilst also providing opportunities for participation:

When I became ill Facebook was a lifeline… (Kate)

Facebook evolved as guiding light for participants who needed to better understand their experience of illness:^25^

Facebook groups help me feel normal and not alone when facing disbelief and lack of support from others. The experiences I have seen from others mirror my own and give me a greater belief in fighting for our rights. (Jessica)

Jessica is demonstrating how engagement affected her identity in a positive way and created new possibilities in acting as an advocate for others. This is echoed by Dan.

I joined a few FB ME groups and began firstly to get an understanding of ME and its implications for myself… Now it is a place where I can help support others coming in new… (Dan)

Engagement in online groups has moved participants from the periphery to full participation, accepting and adapting to their CFS/ME identity. They are also now able to support newcomers, which suggests a renegotiation of their previously stigmatised identities into that of experts, as seen in Lave and Wenger's research within A.A. Gaining a sense of belonging has transformative potential and may contribute to a sense of mastery which has been lost in other roles and communities. This change in identity reflects the possibilities for interrupted trajectories and jeopardised identities to be compensated by the negotiation of virtual trajectories and legitimate CFS/ME identities.

Our participants also gave examples of the ways in which they were able to live alongside their illness by finding ways to participate which further enriched their lives. Through experiences of purpose and meaning they developed opportunities to ‘be’ alongside CFS/ME.

I have taken crochet and knitting back up as I can work at a pace which suits me, I am planning to sell what I make and plan on doing a craft show next year (Netty)

After two and a half years I decided to go to college it makes me feel as if I’m still here if that makes sense (Catriona)

I’ve written many poems about life with M.E. and have recently published a book. I want to raise understanding as well as raise funds for the charity Invest in M.E.

We suggest that adaption and re-engagement in new roles and communities demonstrates the power of renegotiating the stigmatised identity. New identities may be developed which are not centrally defined by loss or stigma. If the CFS/ME community is to be empowered, we argue participation is key. Paying attention to the community practices which promote engagement must be a way forward for individuals, their families and health professionals.

## Conclusion

The application of CoP provided a framework within which to better consider and understand the issues of identity which are often central to the lived experience of chronic illness. Wenger's participation concept underpinned both the grief for a life and self lost in CFS/ME, and participants’ attempt to renegotiate their jeopardised identities.^25^ The application of CoP foregrounded the reality of enabling and disabling communities in CFS/ME. The history of CFS/ME was apparent in the scepticism underpinning the lived experience of the illness for participants and their compromised opportunities to participate. The virtual world became an enabling community which allowed participation to resist stigma and offered ways for them to renegotiate their CFS/ME identities.

Participation in virtual communities may also provide an opportunity for the medical profession(s) to reconsider CFS/ME and contribute towards a shift in the social construction of CFS/ME. As is reflected in the current analysis, the literature argues a central tenet of group participation programmes is the mutual support they offer.^33–35^ This could also be a useful tool in broadening understanding and facilitating a change in practices. For example, group participation programmes involving family members or primary care workers could create a platform for positive change. The CFS/ME community may understandably be dubious of ‘outsiders’ such as health professionals being allowed in, however a commitment such as this could generate understanding in both insiders and outsiders. The importance of supportive communities should not be underestimated when people are struggling to find a new way to be in the world.

## Limitations

Although the insider perspective of the researcher engendered the trust of participants which underpinned their generous contributions, the insider perspective of the researcher may have influenced the interpretation of the data. However, although we acknowledge that the data collection process was undertaken by someone who has experienced CFS/ME personally, for the purpose of this paper, analysis was undertaken independently by each author and a consensus reached about the direction to take. In using participants through Facebook, our sample was limited to those with access and understanding of online communities. Further research is needed on the ways in which community participation can be facilitated in other areas of life to support those with CFS/ME.
